# Cryoablation inhibits the recurrence and progression of bladder cancer by enhancing tumour‐specific immunity

**DOI:** 10.1002/ctm2.1255

**Published:** 2023-05-08

**Authors:** Zezhong Mou, Yiling Chen, Zheyu Zhang, Xinan Chen, Yun Hu, Lujia Zou, Chenyang Xu, Haowen Jiang

**Affiliations:** ^1^ Department of Urology Huashan Hospital Fudan University Shanghai China; ^2^ Fudan Institute of Urology Fudan University Shanghai China; ^3^ National Clinical Research Center for Aging and Medicine Fudan University Shanghai China

**Keywords:** abscopal effect, cryoablation, immune remodelling, pd‐1 inhibitor

## Abstract

**Background:**

Recurrence and metastasis of bladder cancer are major factors affecting patient prognosis. Endoscopic cryoablation achieved a better clinical outcome among clinical patients and could be synergistic with ICIs. Thus, this study aimed to evaluate the immunological mechanism of cryoablation for bladder cancer to reveal the therapeutic mechanism.

**Methods:**

We systematically reviewed the clinical prognosis of patients underwent cryoablation at Huashan Hospital in these first‐in‐human studies (ChiCTR‐INR‐17013060). Murine models were constructed to explore cryoablation‐induced tumour‐specific immunity, which was further confirmed by primary bladder tumour organoids and autologous lymphocytes cocultured system.

**Results:**

Cryoablation improved progression‐free survival and recurrence‐free survival respectively. Assessment of murine models after cryoablation confirmed microenvironment remodelling and tumour‐specific T cells expansion. Enhanced antitumour effects were found after coculture of organoids with autologous lymphocytes collected from post‐cryoablation. We also demonstrated cryoablation‐induced tumour elimination required IFNGR expression on tumour cells. In addition, a long‐lasting antitumour memory response is achieved by cryoablation and could be enhanced after combination with ICIs.

**Conclusions:**

This study revealed endoscopic cryoablation is an efficient and safe therapy for bladder tumour treatment. The tumour‐specific immune responses induced by cryoablation could reduce tumour recurrence and metastasis.

## Graphical abstract

Endoscopic cryoablation eliminated bladder cancer cells and released tumour antigens to induce abscopal effect. Cryoablation remodulated microenvironment immune landscape and induced tumour‐specific T‐cell responses. Tumour‐specific immunity induced by cryoablation was depended on tumour expression of IFNGR. Cryoablation plus ICIs promoted effective long‐term antitumour immunity.

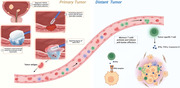

## 1 INTRODUCTION

Bladder cancer is among the top 10 most frequent malignancies in the world, accounting for more than half a million new cases annually.^1^ Approximately 25% of bladder cancers invade the muscular layer at diagnosis and are known as muscle‐invasive bladder cancer (MIBC).^2^ Prevention of tumour recurrence and progression is crucial for patients with early‐stage disease. Intravesical instillation of Bacillus Calmette‐Guerin (BCG) and treatment with immune checkpoint inhibitors (ICIs) played important roles in the treatment of bladder cancer.^3^ However, 30%–50% of patients with nonmuscle‐invasive bladder cancer (NMIBC) show no clinical response to BCG, and 10%–15% progressed to MIBC.^4^ Similarly, ICIs show therapeutic responses in only 20% of patients, which hinders their practical application.^5^, ^6^

Cryoablation is an ablative treatment based on extreme hypothermia that induces necrosis and apoptosis of living cells, and is associated with less surgical trauma than conventional surgical resection.^7^ Hypothermia‐induced necrosis has the potential to elicit systemic antitumour immunity due to the release of tumour antigens, suggesting that cryoablation may function as an autologous tumour vaccine.^8^, ^9^ However, there is limited evidence regarding the use of cryotherapy in treating intraluminal tumours (e.g., bladder cancer), as the existing clinical applications mainly target solid organ malignancies such as the prostate,^10^, ^11^ liver^12^, ^13^ and breast.^14^ Therefore, we developed a novel operative technique called endoscopic balloon cryoablation (EBCA) to treat bladder tumours. The feasibility and safety of EBCA have been confirmed in animal models and clinical trials.^15^, ^16^ Herein, we retrospectively analysed the prognosis of patients with bladder cancer who underwent cryoablation and compared it with that of patients who underwent transurethral resection of bladder tumour (TURBT). We present the first intensive investigation comparing the changes in peripheral blood lymphocyte subsets before and after cryoablation to reveal systemic immunomodulatory activities in patients with bladder cancer. Although this mechanism of antitumour activity is plausible during cryoablation, direct evidence of cryoablation‐induced antigen‐specific antitumour responses is lacking.

To address this knowledge gap, subsequent studies of tumour‐infiltrating lymphocytes (TILs) using a C57 mouse model, including sequencing analysis, T‐cell receptor (TCR) detection, and flow cytometry, have revealed alterations in the TCR library, increased activation of antigen‐specific T lymphocytes as well as secretion of antitumour effector molecules (such as IFN‐γ and Granzyme B) following tumour rechallenge after cryoablation. The prediction that T cells could develop more potent antitumour effector functions due to cryoablation was validated using in vitro and in vivo experimental models, including organoids and adoptive cellular immunotherapy (ACT). In particular, we observed a markedly elevated antitumour effect of T cells in peripheral blood after cryoablation compared to pre‐cryoablation T cells when cocultured with autologous primary cancer organoids. Moreover, a higher level of IFN‐γ was detected in supernatants collected from the coculture system after cryoablation. When IFNGR was knocked out, the specific antitumour immunity induced by cryoablation disappeared. We hypothesised that cryoablation‐induced antitumour immunity depends on IFN‐γ signalling. This phenomenon was verified in tumour microarrays (TMAs), as patients with low IFNGR expression are prone to relapse. Finally, the synergistic effect of combined cryoablation and anti‐PD‐1 treatment was demonstrated.

The present study revealed the dual function of cryoablation in eliminating orthotopic tumour cells and inducing changes in systemic immunologic memory and other immunological effects, which prevent the progression and recurrence of the tumour in patients with bladder cancer.

## 2 MATERIALS AND METHODS

### 2.1 Sample collection and processing

Tissue samples from two cohorts of patients with bladder cancer were collected at Huashan Hospital. Patients in cohort 1 were enrolled in a randomised clinical trial that evaluated the safety and feasibility of EBCA in patients with NMIBC or T2a MIBC (registration number: ChiCTR‐INR‐17013060). Tissue specimens from these patients were collected during diagnostic transurethral resection before endoscopic cryoablation. Tissue detachment from TMAs and immunohistochemistry (IHC) staining analysis were performed as previously described.^17^ Peripheral blood samples were collected on the morning of the surgery, and peripheral blood mononuclear cells (PBMCs) were prepared by density gradient centrifugation, frozen in liquid nitrogen and stored at –80°C until use. Tissue samples and associated clinicopathological information were obtained from the HS‐Cryo database and approved by the Institutional Ethics Committee.

### 2.2 Murine model and study design

All animal studies were approved by the Ethics Committee and performed in accordance with ethical standards. Male wild‐type C57 BL/6J (Phenotek, Shanghai) between 6 and 8 weeks of age were used. A tumour‐bearing mouse model was constructed by subcutaneous injection of the mouse bladder cancer carcinoma cell line MB49 (5 × 10^5^ cells/mouse) into the right armpit of each mouse. After 14 days, the tumour was frozen using a specially designed cryoablation unit (Patent No. 201510398917.6) for two cycles, with each cycle involving 40‐second freezing and 90‐s thawing. No treatment was administered to mice in the blank group. The control group underwent surgical resection of the primary tumour. The mice were anesthetised with isoflurane, and strict aseptic conditions were maintained during surgery. The surgical sites were sterilised twice a day after surgery, and the bedding was changed daily to prevent infection. On the second day after surgery, MB49 cells (5 × 10^5^ cells/mouse) were reimplanted in the contralateral armpit. The size and volume of the secondary tumours were observed and growth curves were plotted. The experimental endpoint was defined as death or a secondary tumour volume ≥ 2000 mm^3^.

### 2.3 Cell culture

MB49 murine urothelial tumour cell was obtained from Millipore Corporation (Millipore, MD, USA) and B16F10 melanoma cell line was purchased from ATCC. MB49 and B16F10 cells were placed in complete DMEM (GE Healthcare Life Sciences, Pittsburgh, PA, USA) and RPMI‐1640 medium (Gibco; Thermo Fisher Science, Massachusetts, USA) medium containing 10% fetal bovine serum (FBS; Gibco; Thermo Fisher Science, Massachusetts, USA), 1% penicillin/streptomycin at 37°C in a 5% CO_2_ humidified incubator. Luciferase‐expressing (MB49‐luc) was generated by transfection with lentiviral vectors (Genomeditech, Shanghai, China) encoding for firefly luciferase. All cells were tested for Mycoplasma (MycoAlert Mycoplasma Detection Kit, Lonza Group LTD) and retested prior to use.

### 2.4 TCR repertoire analysis

Fastq files generated from RNA‐sequencing were processed to quantitate clonotypes using MiXCR software,^18^ which can efficiently handle paired‐ and single‐end reads, consider sequence quality, correct PCR errors and identify germline hypermutations. Quantitative clonotype data were acquired using the default packed shotgun analysis pipeline of MiXCR. Clonotypes, CDR3, clonality and diversity estimation were performed and visualised using the R package immunarch.

### 2.5 IHC staining

IHC staining was performed using dewaxed tissue slides. After hydration, tissue slides were heated in citrate buffer (10 mM, pH 6.0) to extract antigens and 3% H_2_O_2_/MeOH to inhibit endogenous peroxidase. To block nonspecific binding sites, the slides were implanted with a blocking buffer, including 10% horse serum. The slides were then exposed to primary antibodies in a wet chamber at 4°C for 16 h and treated with a biotin‐labelled goat anti‐mouse/rabbit IgG secondary antibody. The excess antibody was removed by washing thrice with PBS and visualised using the DAB Substrate Kit until the desired brown colour was achieved. Slides were scanned using Pannoramic SCAN II.

### 2.6 Single‐cell separation and flow cytometry analysis

The mice were anaesthetised and euthanised at predetermined time points. For the analysis of lymphocyte subsets and function in the tumour microenvironment, the secondary tumours were isolated, weighed and treated with RPMI‐1640, 5% FBS, collagenase IA (1 mg/mL, Sigma‐Aldrich), and DNase I (0.25 mg/mL, Sigma‐Aldrich) at 37°C for 45 min, while splenic lymphocytes were extracted by grinding. Subsequently, the digested and splenic lymphocytes were filtered through 70 µm cell strainers and centrifuged at 300 *g* for 10 min. After resuspension, red blood cells were lysed using the Red Blood Cell Lysis Buffer (Yeason Biotechnology, Shanghai). To investigate tumour‐related effector molecules, 1 × 10^6^ cells were incubated in RPMI‐1640 with 5% FBS and Cell Stimulation Cocktail (plus protein transport inhibitors, eBioscience, 500×) at 37°C. After 4 h, the cells were harvested and analysed further. Information regarding the antibodies used for flow cytometric analysis is listed in Table S2. The protocol for flow staining has been described previously.^19^ Flow cytometry data were analysed using FlowJo software (Version 10.8.1, FlowJo, LLC).

### 2.7 Tumour organoid isolation and coculture

The protocol for culturing organoids in vitro has been reported previously.^20^ Briefly, tumour tissues from patients were washed in organoid medium for 5 min, cut with pre‐sterilised microdissection scissors, placed in 10 mL of organoid medium, and digested with collagenase/trypsin (2 mg/mL) at 37°C with 5% CO_2_ for 45 min. Isolated tissues were centrifuged at 350 *g* for 5 min at room temperature. The supernatant was discarded, and the precipitate was resuspended in 60% Matrigel matrix (Basement Membrane Matrix, Corning). Following this, 250 µL of the mixture was added to each well of a six‐well plate and incubated at 37°C with 5% CO_2_ for 30 min. Once the Matrigel solidified, 2 mL of organoid medium was added to each well and changed regularly every 3 or 4 days. Tumour organoids with good activity after three to six generations were isolated from Matrigel, dissociated into single cells, and cocultured in vitro with autologous T cells collected before and after cryoablation at a 1:10 ratio. T‐cell separation was performed as previously described.^19^ After coculture for 48 h, the tumour cells were isolated for flow cytometry to calculate the proportion of dead 7‐AAD‐positive cells. Cytokines in the supernatants were collected for detection using an ELISA kit (Elabscience Biotechnology, Wuhan, China), according to the manufacturer's instructions.

### 2.8 In vivo injection of neutralising antibodies

CD4^+^ T lymphocytes and CD8^+^ T lymphocytes were depleted by intraperitoneal injection of 200 µg neutralising antibody (InVivoMab anti‐mouse CD8α, Bio X Cell, Clone: 2.43; or InVivoMab anti‐mouse CD4, Bio X Cell, Clone: GK1.5) on the day of cryoablation and every 3 days from the second day after cryoablation. The depletion efficacy was verified by flow cytometry. Anti‐mouse PD‐1 (200 µg/dose, InVivoMab anti‐mouse PD‐1, Bio X Cell, Clone: J43) was injected into rechallenged mice after cryoablation or surgical resection of primary tumours every 3 days for a total of five times.

### 2.9 Adoptive cell transfer therapy

The spleens of mice after cryoablation or tumour resection were collected, ground and filtered through a 70 µm cell strainer. T cells were purified using the Pan T‐cell isolation kit (Miltenyi Biotec) before being seeded into six‐well plates at 1 × 10^5^ cells/well. RPMI 1640 (Gibco) was added with anti‐mouse CD3/CD28 (5 µg/mL) and IL‐2 (2000 IU/mL) for cell culture and expansion. MB49‐Luc cells were subcutaneously injected (3 × 10^6^ cells/mouse) into the right armpit of 6‐week‐old male NPSG mice (NOD‐Prkdc^scid^IL2rg^null^, Phenotek, Shanghai). When a small nodule could be palpated under the skin about 3 days after implantation, activated T cells (5 × 10^6^ cells) were injected via the tail vein into the mice for ACT. On days 3, 6, 9, 14 and 18, the mice were imaged using intravital imaging. The efficacy of ACT was evaluated by comparing the volume and fluorescence intensity of the tumours.

### 2.10 CRISPR/Cas9

Knockout of the IFNGR gene in the MB49 cell lines via the CRISPR/Cas9‐gDNA system was accomplished by Genomeditech (Shanghai, China). The gRNA sequence for IFNGR is TCGCGCAGGAATGGGCCCGC. The IFNGR knockout cell line was selected by flow cytometry analysis, which based on functional testing by adding recombinant mouse IFN‐γ (Novoprotein, China)  at a concentration of 100ng/mL for 48 h.

### 2.11 RNA extraction and qRT‐PCR analysis

Total RNAs were extracted from tissues by using Trizol (Invitrogen, USA) following the manufacturer's instruction as previously described.^21^ Reverse transcription was performed by using Hifair® III 1st Strand cDNA Synthesis SuperMix for qPCR (Yeason Biotechnology, Shanghai) to obtain cDNA. RT‐qPCR was performed by using Hieff UNICON® Universal Blue qPCR SYBR Green Master Mix (Yeason Biotechnology, Shanghai) on an ABI 7900HT sequence detection machine (Thermo Fisher Scientific, Massachusetts, USA). GAPDH RNA was used as an internal control. The 2^−ΔΔCT^ method was used to calculate different expression of RNAs. The experiments were repeated three times. All primers used were listed in Table S3.

### 2.12 Statistical analysis

All statistical analyses were performed using GraphPad Prism 9 (Version 9.4, GraphPad Software, CA, USA). Survival analysis was performed using the Kaplan–Meier method, and survival differences were compared using the log‐rank test. For sequencing analysis, comparisons between two groups were performed using unpaired Student's *t*‐tests. One‐way or two‐way ANOVA tests were used to determine *p* value. The data were analysed using Student's *t*‐test when normally distributed, or using nonparametric tests if not. Statistical significance was set at *p* < .05.

## 3 RESULTS

### 3.1 Cryoablation increased antitumour protection in both patients and murine model

**FIGURE 1:**
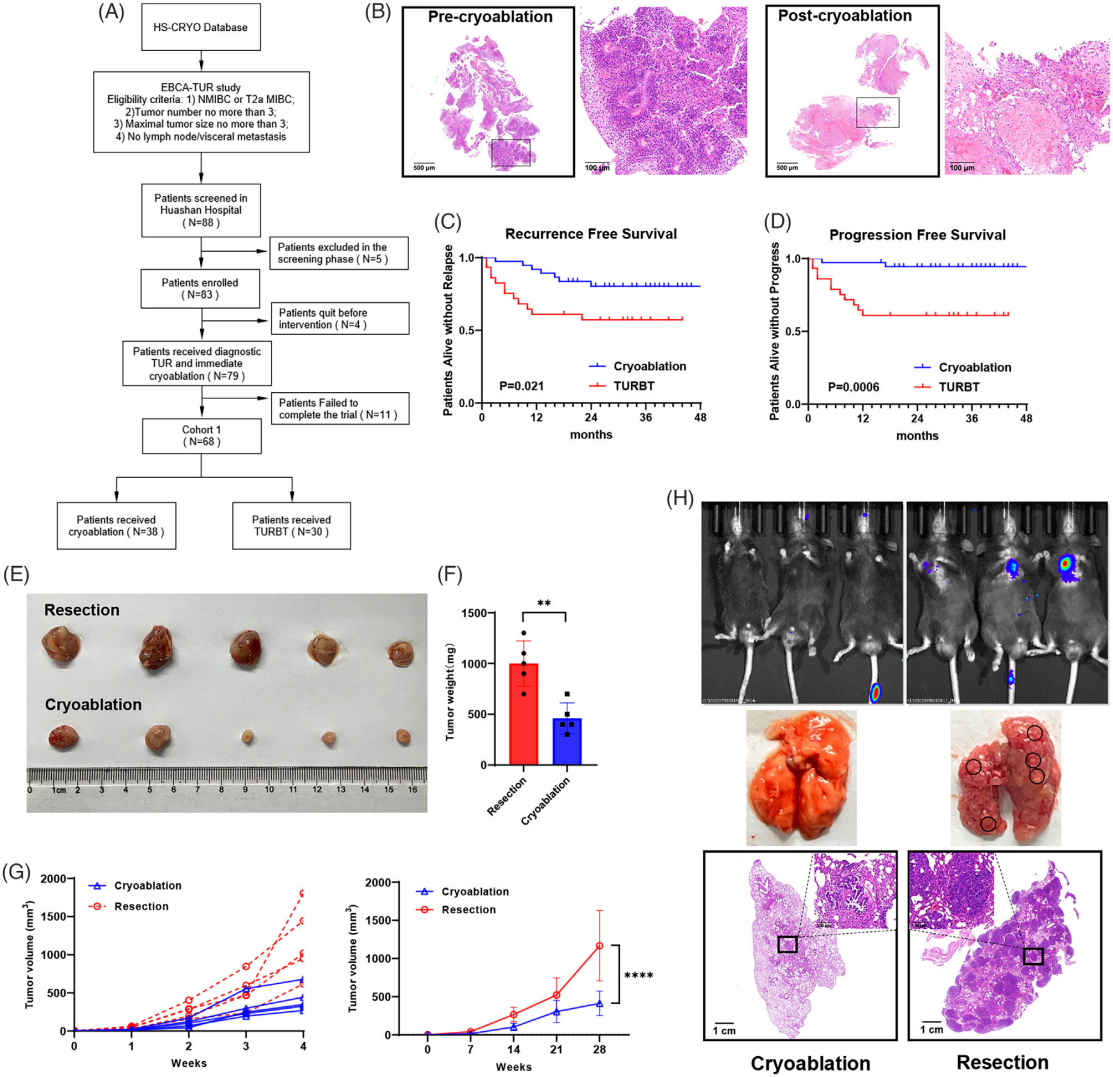
Cryoablation reduced the recurrence and progression of bladder cancer. (A) Flowchart of the clinical trial of cryoablation for bladder cancer. (B) Representative IHC results of tumour tissues surgically excised before cryoablation and tissues in situ at the 3‐month review after cryoablation from the same patient. Scale bars, 500 µm (4×), 100 µm (20×). (C) Kaplan–Meier curves for recurrence‐free survival of patients treated with EBCA or TURBT (*p* = .021). (D) Kaplan–Meier curves for progression‐free survival of patients treated with EBCA or TURBT (*p* = .0006). (E, F) Secondary tumours were removed and measured after rechallenge with 5×10^5^ MB49 cells on the second day after cryoablation or resection of the primary tumours. *n* = 5 mice per group. Data are represented as mean ± SD. **p < .01, cryoablation vs. resection. (F, G) The volume of the secondary tumours was dynamically observed once per week after rechallenge with 1×10^5^ MB49 cells until death or until the secondary tumour volume reached 2000 mm^3^. *n* = 5 mice per group. ****p < .0001, cryoablation vs. resection. (H) In vivo imaging and HE staining of metastatic foci in the lungs of mice injected with 3×10^5^ MB49‐Luc cells intravenously after cryoablation or surgical resection of the primary tumours. Imaged and evaluated on day 21. *n* = 3 mice per group. Scale bars, 1 cm (1×), 100 µm (20×).

Cryoablation of bladder cancer can lead to the release of tumour antigens during tumour cell necrosis and can induce systemic antitumour immunity with abscopal effects. From November 2017 to September 2020, 218 patients were recruited from six centres for the EBCA‐TUR study (Trial registration number: ChiCTR‐INR‐17013060). The study met its primary outcome with a local control rate of 91.5% in the cryoablation group compared to 76.5% in the TURBT group. Of these patients, 88 were screened for eligibility at the hospital, and 68 patients completed the trial (Table S1). The trial process is illustrated in Figure 1A. Biopsy specimens were collected for IHC staining from the same area of the same patient before and three months after cryoablation during follow‐up to verify the efficacy and safety of EBCA in eliminating bladder tumours. It was confirmed that tumour cells were eliminated after cryoablation, with scar formation and increased lymphocytic infiltration (Figure 1B). No bladder perforation was observed (Figure S1A). Follow‐up results (follow‐up time ranged from 15 to 50 months, and the median follow‐up time was 31 months) revealed that the survival of patients in the cryoablation group was promising, with a 2‐year progression‐free survival (PFS) of 94.7% and 63.3% (cryoablation VS TURBT, *p* = .0006), and a 2‐year recurrence‐free survival (RFS) of 81.6% and 60.0% (cryoablation VS TURBT, *p* = .021), respectively (Figure 1C and D). To comprehensively investigate the antitumour effects of cryoablation, we constructed a murine model of bladder cancer treated with EBCA (Figure S1B). The cellular structure of the tumour was destroyed after treatment, confirming the efficacy of cryoablation (Figure S1C). The tumour volume and weight were both significantly smaller in the cryoablation group after tumour rechallenge (Figure 1E–G). Kaplan–Meier survival curves showed longer survival times in tumour‐bearing mice after cryoablation, demonstrating the abscopal inhibition of tumour growth after cryoablation (Figure S1D). We also observed that almost no lung metastasis occurred after rechallenge with MB49‐luc tumour cells via the tail vein of mice after cryoablation (Figure 1H). These results strongly suggest that cryoablation has the potential to effectively reduce abscopal tumour relapse and metastasis.

### 3.2 Cryoablation remodulated the immune landscape and promotes T‐cell infiltration

**FIGURE 2:**
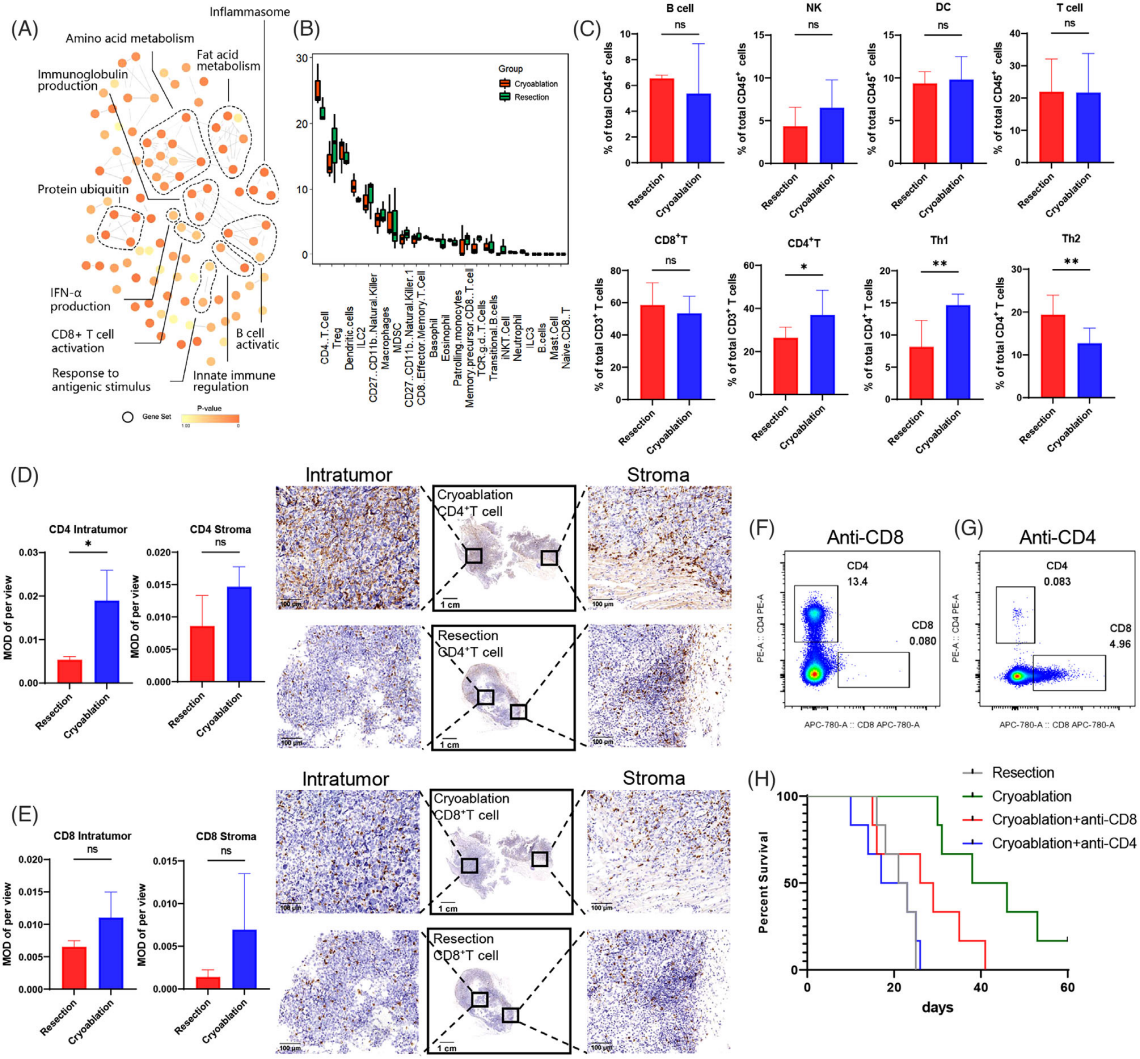
Cryoablation modulated the tumour immune microenvironment. (A) Network of molecular pathways of RNA‐sequencing experiments conducted on TILs isolated from mice after cryoablation or resection. (B) Immuno‐infiltration analysis of the transcriptome using CIBERSORT. Orange represents the cryoablation group and green represents the resection group. (C) Flow cytometric analysis of lymphocyte subpopulations isolated from secondary tumours in the cryoablation and the resection groups. Data are represented as the mean ± SD. **p < .01, *p < .05, cryoablation vs. resection. (D, E) Immunohistochemical staining of CD4^+^ and CD8^+^ T cells in the secondary tumours in cryoablation and resection groups to compare the total number of T cells and infiltrated T cells inside or at the margin of the tumours. Data are represented as the mean ± SD. *p < .05, cryoablation vs. resection. Scale bars, 1 cm (1×), 100 µm (20×). (F) Flow cytometry validating the depletion efficacy of CD8^+^ T cells in a murine model. (G) Flow cytometry validating the depletion efficacy of CD4^+^ T cells in a murine model. (H) Kaplan–Meier curves showing the overall survival of rechallenged mice after different treatments for primary tumours: surgical resection, cryoablation, cryoablation plus depletion of CD4^+^ T cells, and cryoablation plus depletion of CD8^+^ T cells.

To determine changes in the microenvironment after cryoablation, TILs from secondary tumours were isolated, and transcriptome sequencing analysis was performed. Functional enrichment analysis revealed alterations in multiple cell metabolic pathways and immune regulation after cryoablation (Figure 2A). Additionally, we performed immune infiltration analysis using CIBERSORT to draw a preliminary conclusion that T cells are associated with cryoablation (Figure 2B). Changes in immune cell subsets were further verified by flow cytometry (Figure 2C). The total gating strategy for flow cytometry showed in the Figure S2. Unexpectedly, we found an elevated proportion of immunosuppressive cell subsets (Tregs and MDSCs) within the tumour that was rechallenged after cryoablation (Figure S3A and B). We then dynamically examined the changes after cryoablation or resection without tumour rechallenge. Treg cells and MDSCs were elevated in the cryoablation group shortly after surgery. Over time, the proportion of Treg cells tended to decrease (Figure S3C), while the proportion of MDSC cells increased and remained at a low level (Figure S3D). IHC staining of the secondary tumours revealed cryoablation promoted the infiltration of CD4^+^ and CD8^+^ T cells into the tumours. In particular, the number and proportion of CD4^+^ T lymphocyte infiltration increased significantly (Figure 2D and E). To clarify which type of T cells played a predominant role in the antitumour response, CD4^+^ or CD8^+^ T cells were depleted during the tumour rechallenge phase (Figure 2F and G). When only CD4^+^ T cells were present, the prognosis was improved compared to that of CD8^+^ T cells alone; however, there was no significant advantage compared to the groups without depletion (Figure 2H). Therefore, antitumour effects induced by cryoablation require synergy between CD4^+^ and CD8^+^ T cells. These data suggest that cryoablation could remodel the immune microenvironment and increase the infiltration of lymphocytes inside the tumour, switching it into immune‐active and ‘hot’ microenvironments.

### 3.3 Cryoablation‐induced tumour‐specific T‐cell responses

**FIGURE 3:**
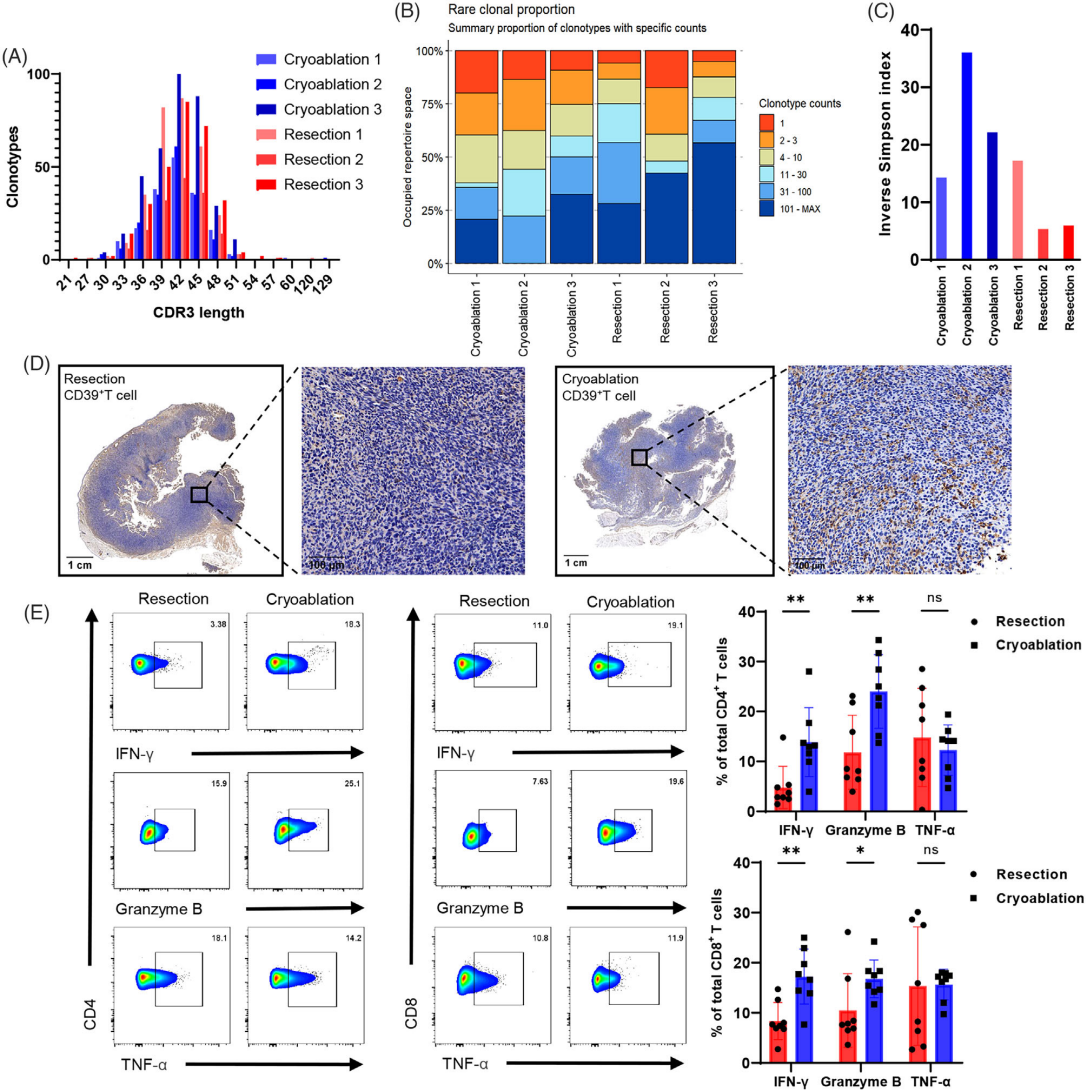
Cryoablation promoted the expansion of tumour‐specific T cells. (A) Distribution of abundant TCRB CDR3 clonotypes after cryoablation and resection. (B) Proportion of rare clonal populations. The bars represent the summary proportions of the clonotypes with specific counts. (C) Inverse Simpson index values in the cryoablation and resection groups. (D) IHC for CD39 as a marker to distinguish tumour‐specific T cells from bystander T cells. Scale bars, 1 cm (1×), 100 µm (20×). (E) Flow cytometric analysis of the differences in the secretion of tumour effector molecules IFN‐γ, Granzyme B and TNF‐α by CD4^+^ and CD8^+^ T cells after cryoablation (*n* = 8) or surgical resection (*n* = 8). Data are represented as the mean ± SD. *p < .05, **p < .01, cryoablation vs. resection.

Generally, increased lymphocytic infiltration in the tumour microenvironment suggests a better prognosis. However, there is a certain percentage of T cells in the microenvironment, known as bystander T cells,^22^ which are tumour‐ignorant and cannot exert antitumour activity. Therefore, we designed the following experiments to further verify whether the contents released from fragmented tumour cells after cryoablation could act as tumour‐associated or ‐specific antigens and activate specific antitumour immunity. MB49 and melanoma cells B16F10 were chosen to subcutaneously inoculate C57 mice under the armpits followed by cryoablation or resection and B16F10 rechallenge (Figure S4A). Tumour growth and metastasis were not inhibited after B16F10 rechallenge in mice implanted with MB49 (Figure S4B–D). In fact, neither surgical resection nor cryoablation in the MB49 groups could prevent B16F10 rechallenge, suggesting that the aforementioned immune response induced by cryoablation might be tumour‐specific rather than treatment‐specific. Further TCR‐sequence analysis of TILs revealed that the length of CDR3 changed after cryoablation (Figure 3A), and the rare clonal proportion increased (Figure 3B), suggesting that some tumour‐specific T cells might have expanded after cryoablation. The Simpson index also decreased after cryoablation, which represented an increase in the abundance of the TCR library (Figure 3C). We also analysed the diversity of TCRs using a different index, and the results revealed an increase in clonal subpopulations of T cells after cryoablation (Figure S5A and B). Previous studies have demonstrated that CD39 serves as a reference indicator to distinguish tumour‐specific T cells from bystander T cells.^23^ The IHC results of our experiments revealed upregulated CD39 expression after cryoablation, as well as an increased number in the tumour microenvironment and a trend towards deeper infiltration of CD4^+^CD39^+^ and CD8^+^CD39^+^ T cells (Figure 3D and Figure S5C and D). Moreover, proportion of activated T cells were dynamically detected at 0, 24, 48 and 72 h after cryoablation or resection. Early active CD69^+^ T cells were at a low expression level before cryoablation and rapidly increased within 24 h after cryoablation, while CD25^+^ T cells increased slowly after cryoablation and showed significant changes at 72 h (Figure S6A and B). Expression of T cells activation related transcription factors also elevated early after cryoablation (Figure S6C). Subsequent analysis of effector molecules in the tumour microenvironment revealed increased secretion of IFN‐γ and Granzyme B after cryoablation (Figure 3E and Figure S6D).

### 3.4 Cryoablation boosted tumour‐specific memory and exerted a long‐lasting immune surveillance

**FIGURE 4:**
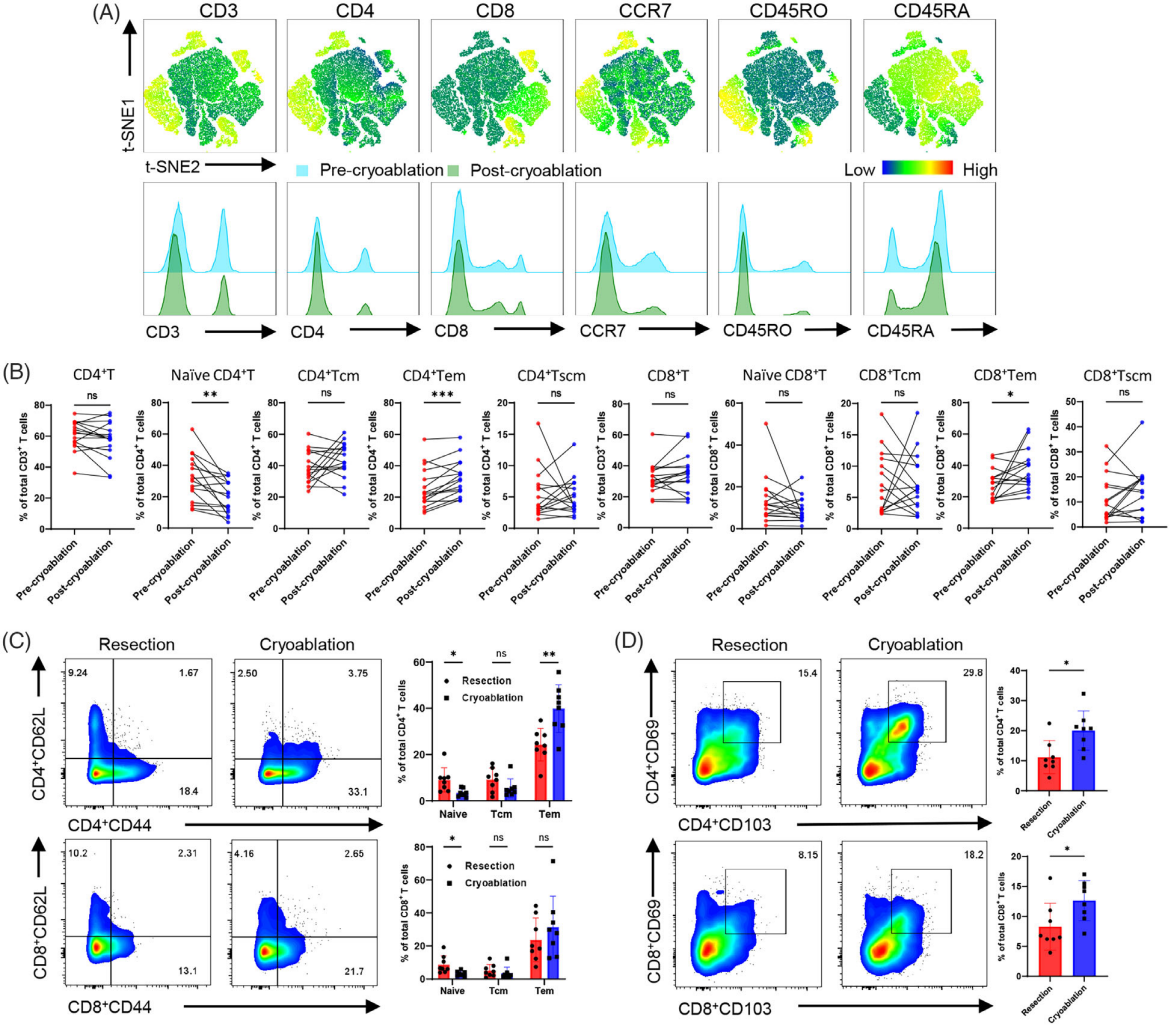
Cryoablation boosted tumour‐specific immune memory. (A) T‐SNE maps showing the different immune lineages of memory T cells separated from the autologous PBMC of patients with bladder cancer. Differences in immune lineages are shown as a histogram. (B) Paired column plots illustrating changes in CD4^+^/CD8^+^ T cells and naïve T, T_cm_, T_em_ and T_scm_ cells in the same individual (*n* = 16) before and after cryoablation. *p < .05, **p < .01, ***p < .001, post‐cryoablation vs. pre‐cryoablation. (C) Flow cytometry showing the proportion of CD4^+^ and CD8^+^ memory T lymphocytes in the secondary tumours of mice in the cryoablation group (*n* = 8) and resection group (*n* = 8) after rechallenge. CD62L^+^CD44^−^, naïve T cells; CD62L^+^CD44^+^, central‐memory T cells; CD62L^−^CD44^+^, effector‐memory T cells. Data are presented as mean ± SD, *p < .05, **p < .01, cryoablation vs. resection. (D) Proportion analysis of the tumour‐infiltrating CD4^+^ and CD8^+^ tissue‐resident memory T cells. Data are presented as the mean ± SD, *p < .05, cryoablation vs. resection.

Most T cells undergo apoptosis after performing their effector functions, whereas the remaining cells differentiate into long‐lived memory T lymphocytes that can activate a strong secondary immune response. Therefore, we performed a flow cytometric analysis of memory T‐cell subsets in patients with bladder cancer after cryoablation. We isolated and identified memory T‐cell subsets in autologous PBMC before and 3 months after cryoablation (Figure 4A). Unexpectedly, we observed a decrease in the ratio of CD4^+^ naïve T cells. In contrast, both proportions of CD4^+^ T_em_ and CD8^+^ T_em_ cells were significantly upregulated (Figure 4B). To explore the potential patterns of these changes, the tumour and splenic lymphocytes in the rechallenged mice were further analysed. Naïve T cells and CD44^+^CD62L^+^ T_cm_ cells were significantly reduced after cryoablation, whereas CD44^+^CD62L^−^ T_em_ cells were increased in both tumours and spleens (Figure 4C and Figure S7A and B). Previous studies have shown that an elevated T_cm_/T_em_ cell ratio is associated with a lower postoperative recurrence rate and better prognosis for patients.^24^ Therefore, we dynamically monitored memory T‐cell subsets in mice after cryoablation and surgical removal of in situ tumours. The number of T_cm_ cells was found to increase and subsequently decrease in the short term after resection or cryoablation, and an increased proportion of T_cm_ cells was observed after 14 days in the cryoablation group. The proportion of T_em_ cells also increased after cryoablation, and the ratio of T_cm_ to T_em_ cells increased with extended observation time (Figure S7C–E). Tumour‐associated tissue‐resident memory T cells (T_rm_) are a group of T cells residing in the tumour microenvironment with both effector and memory T‐cell characteristics that perform both immunosurveillance and antitumour functions. In our study, elevated expression of CD103^+^CD69^+^ T_rm_ cells was observed after rechallenge in the cryoablation group (Figure 4D), and the results of immunofluorescence analysis suggested more intratumoural infiltration of T_rm_ cells (Figure S7F and G). These observations indicate that cryoablation induced the expansion of memory T cells, which could generate strong secondary immune responses against tumour recurrence and progression when presented with tumour antigens.

### 3.5 Cryoablation‐induced tumour‐specific immunity was more effective and depended on tumour expression of IFNGR

**FIGURE 5:**
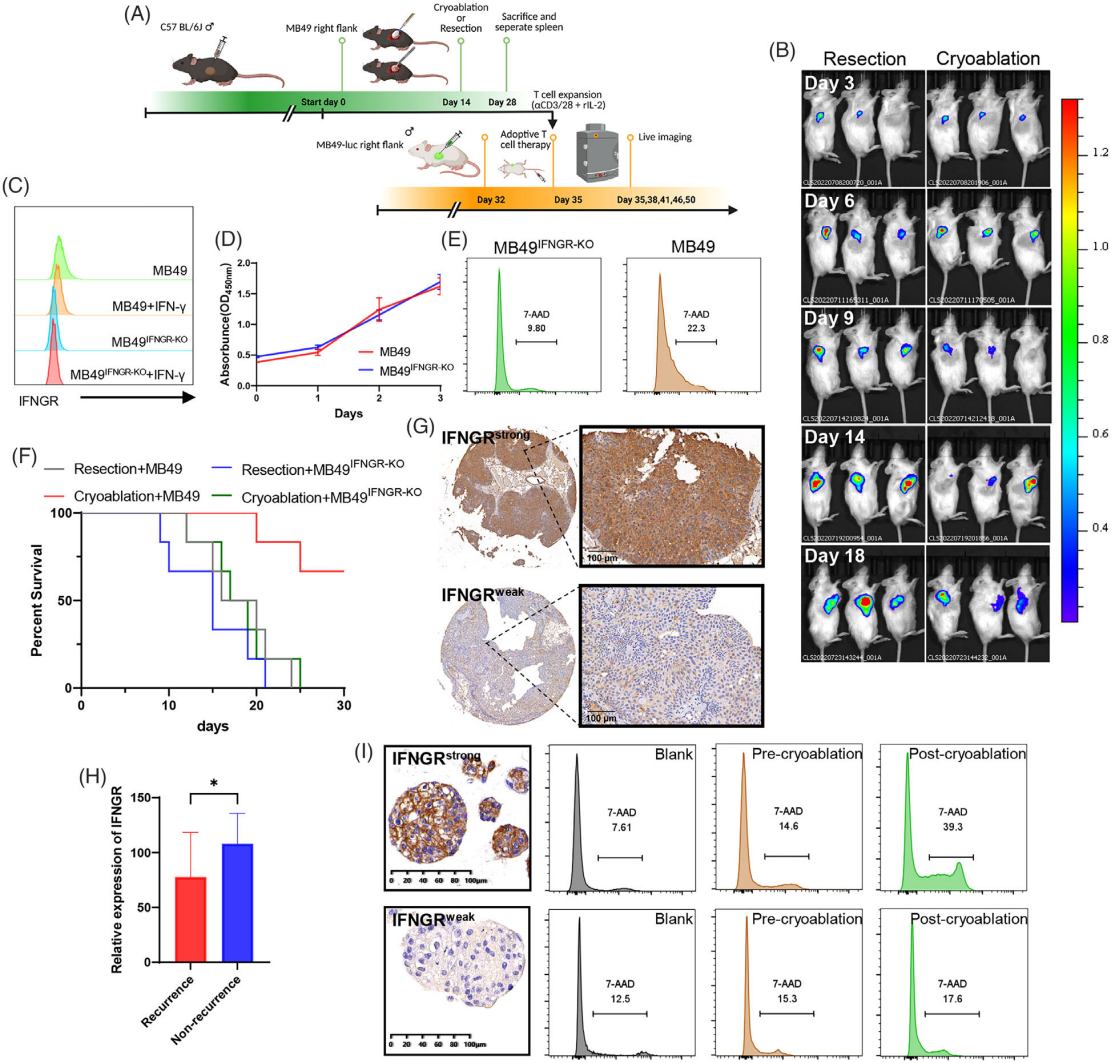
Cryoablation promoted the antitumour ability of tumour‐specific T cells and induced antitumour immunity that required IFNGR expression. (A) Schematic representation of experimental design. (B) Live imaging recording changes in subcutaneous tumours in immunodeficient mice on days 3, 6, 9, 14, and 18 after ACT via intravenous injection of activated and expanded T cells in vitro. (Left) T cells from the resection group. (Right) T cells from the cryoablation group. (C) Flow cytometric analysis of IFNGR expression in MB49, MB49^IFNGR‐KO^ and with or without IFN‐γ stimulation (D) CCK‐8 assay showing proliferation of MB49 and MB49^IFNGR‐KO^ (E) MB49 and MB49^IFNGR‐KO^ cocultured with T cells collected after cryoablation. Dead cells were stained with the cell death marker 7‐Aminoactinomycin D (7‐AAD). (F) Kaplan–Meier curves of mice after MB49 or MB49^IFNGR‐KO^ rechallenge (*n* = 6 per group). (G) Representative IHC staining images illustrating intratumoural IFNGR expression based on weak and strong staining intensity. (H) IFNGR expression was lower in patients who relapsed after cryoablation. Data are represented as mean ± SD, *p < .05, nonrecurrence vs. recurrence. (I) Representative IHC staining images showing that primary organoids derived from the tumour tissues of patients who relapsed after cryoablation had weak IFNGR expression compared with nonrelapse patients. Organoids with IFNGR^weak^ and IFNGR^strong^ were cocultured with autologous T cells collected before and after cryoablation. Dead organoids were stained with the cell death marker 7‐Aminoactinomycin D (7‐AAD).

To further investigate whether cryoablation‐induced T cells possessed an enhanced ability to kill tumour cells, CD3^+^T cells from C57 mice were isolated and expanded in vitro two weeks after surgical resection and cryoablation, and a model of subcutaneous tumour‐bearing NPSG mice was constructed. ACT was initiated when a small tumour mass was palpable, which occurred approximately 3 days after inoculation, using 5 × 10^6^ expanded T cells injected intravenously (Figure 5A). Dynamic live imaging revealed T cells transferred from mice that underwent cryoablation exhibited a stronger suppressive effect on implanted tumours (Figure 5B). Subsequently, we collected tumour tissues from puncture biopsies or surgical resections before cryoablation for tumour organoid culture and identified markers of epithelial tumours using IHC (Figure S8A). In addition, we collected autologous T cells before and after cryoablation and cocultured them with organoids for 48 h. Effector cytokines in the supernatants collected from the coculture system were analysed by ELISA. However, only IFN‐γ level was higher after cryoablation and coculture (Figure S8B). Thus, we predicted that cryoablation‐induced antitumour immunity would depend on the IFN‐γ signalling pathway, which is currently understudied. IFNGR was knocked out, and the addition of IFN‐γ resulted in the downregulation of IFNGR expression in MB49, while no change was observed in MB49^IFNGR‐KO^. The contrast indicates the activated status of the IFN‐γ signalling pathway (Figure 5C). We then demonstrated that IFNGR knockout had no impact on cell proliferation (Figure 5D), whereas cell death (7‐Aminoactinomycin D [7‐AAD] positive) of MB49^IFNGR‐KO^ was reduced when cocultured with tumour‐specific T cells from post‐cryoablation mice (Figure 5E). The abscopal antitumour effect of cryoablation was also lost when mice were rechallenged with MB49^IFNGR‐KO^, and the prognosis worsened (Figure 5F). Next, we examined IFNGR expression in TMAs from cryoablation treated patients (Figure 5G), and found significantly lower expression in patients with tumour relapse (Figure 5H). Additionally, the tumour cell‐killing ability of T cells was weakened in patients with low IFNGR expression in their tumour organoids (Figure 5I). The aforementioned results indicate that the expression of IFNGR in tumour is required for cryoablation‐induced antitumour immunity, and is positively associated with better prognosis after cryoablation.

### 3.6 Cryoablation plus PD‐1 inhibitor treatment promoted effective long‐term antitumour immunity

**FIGURE 6:**
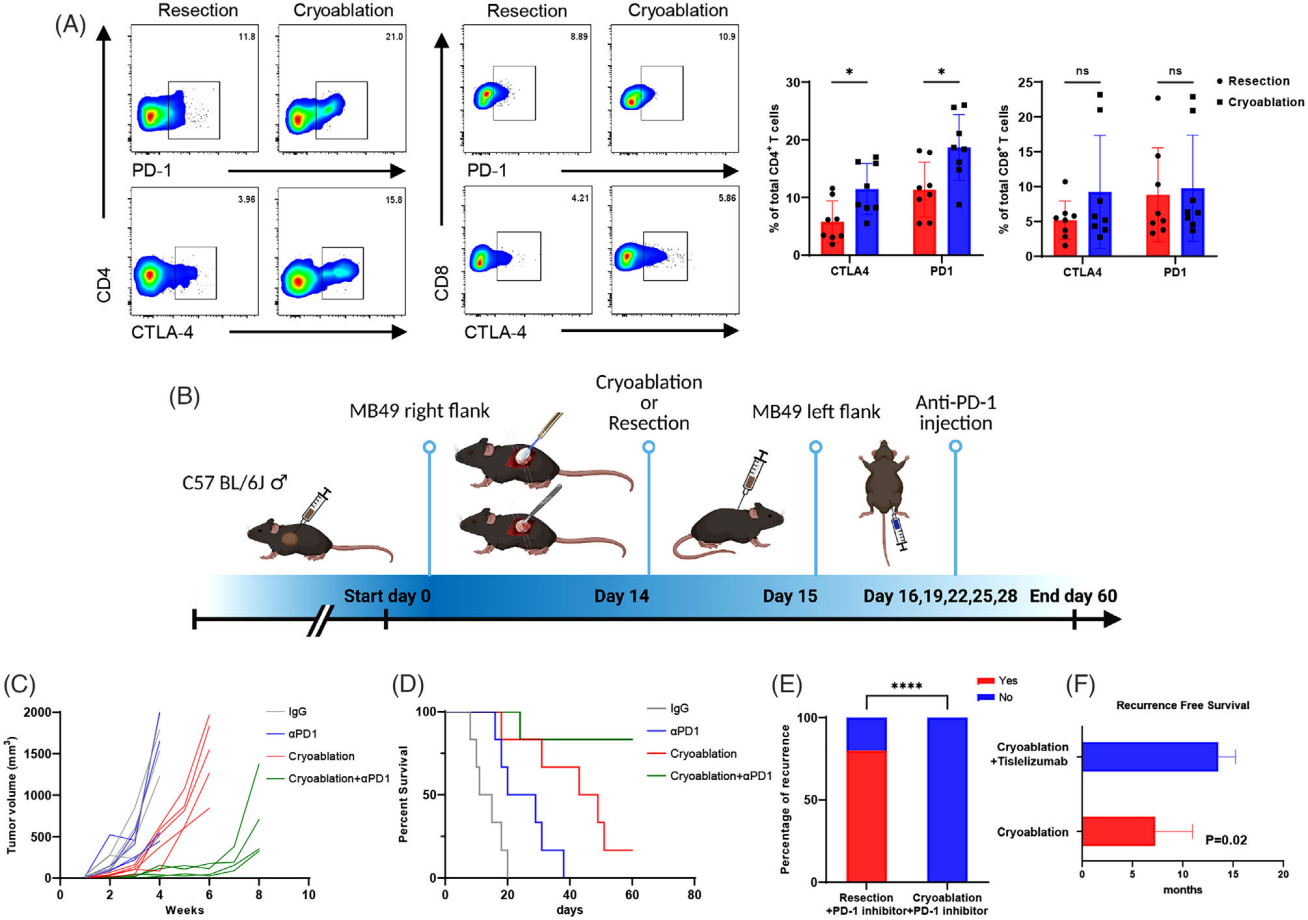
Cryoablation and PD‐1 inhibitors exerted synergistic antitumour effects. (A) Flow cytometric analysis of PD‐1 and CTLA‐4 expression in CD4^+^ and CD8^+^ T cells of secondary tumours after cryoablation (*n* = 8) or surgical resection (*n* = 8). Data are represented as the mean ± SD, *p < .05, cryoablation vs. resection. (B) Schematic diagram of the evaluation of the combination therapy. (C) Volume growth curve of secondary tumours in each group (IgG, αPD1 only, cryoablation only and cryoablation+αPD1). *n* = 5 mice per group. Observed every week until death or until the tumour volume reached 2000 mm^3^. (D) Kaplan–Meier curves illustrating the survival of mice after rechallenge (*n* = 6 per group). (E) Occurrence of secondary tumours using 5 × 10^5^ MB49 cells 30 days after the combination therapy of cryoablation/resection and αPD‐1. ****p < .0001, cryoablation+PD‐1 inhibitor vs. resection+ PD‐1 inhibitor. (F) Recurrence‐free survival of patients with bladder cancer treated with cryoablation alone and cryoablation in combination with tislelizumab. *p* = .02, cryoablation + tislelizumab vs. cryoablation.

Given that T cells are exhausted after activation, we investigated whether anti‐PD1 improves cryoablation‐induced tumour‐specific T cell function. The immune checkpoints PD‐1 and CTLA‐4 were significantly upregulated in the rechallenged tumours after cryoablation (Figure 6A and Figure S6E). We then constructed an animal model based on the combination therapy (Figure 6B) and found that it resulted in long‐term maintenance of immune balance, and the application of the PD‐1 inhibitor alone was associated with a better prognosis (Figure 6C). However, the tumour volume did not decrease significantly; in fact, it increased compared with the vehicle group, which could be explained by the occurrence of pseudo‐progression (Figure 6D). We also selected five pairs of mice after cryoablation or resection, treated them with a PD‐1 inhibitor, and rechallenged MB49 cells on day 30. Four mice from the resection group developed recurrence after receiving the same PD‐1 inhibitor treatment, whereas those in the cryoablation group did not develop any recurrence (Figure 6E). In addition, we collected 27 patients with high‐risk NMIBC or patients with T2‐T3 MIBC for evaluation of cryoablation‐based bladder sparing strategies. Among them, 13 patients received at least six courses of tislelizumab after cryoablation. The RFS of patients with relapse was prolonged (*p* = .02) in the group that received a combination of cryoablation and tislelizumab compared with those who underwent cryoablation alone (Figure 6F). Taken together, the results from both animal experiments and clinical trials indicated a synergistic relationship between cryoablation and PD‐1 inhibitors, which could reduce tumour recurrence and progression.

## 4 DISCUSSION

With the recent development of immunotherapy, ICIs have provided new choices for bladder cancer treatment. Current guidelines have recommended ICIs, represented by PD‐1/PD‐L1 inhibitors, for patients with advanced metastatic bladder cancer that has progressed after chemotherapy; however, the effectiveness of these therapies is not notable.^5^, ^25^ Endoscopic cryoablation has emerged in recent years as a surgical modality for the treatment of intraluminal malignancies. Unlike thermal ablation, endoscopic cryoablation uses extreme hypothermia to kill tumours, causing tumour cell necrosis rather than apoptosis,^26^ preserve protein structure and release tumour antigens to activate tumour‐specific T‐cell immunity.^27^ The clinical application of cryoablation has been largely restricted to solid tumours in the liver, breast, kidney, skin, and other solid organs.^28^, ^29^, ^30^ Currently, the most commonly used equipment is the argon‐helium cryoablation system, which is designed with a cryoprobe to puncture and freeze the tissue within and around the tumours.^31^, ^32^ However, percutaneous cryoablation devices are not suitable for treating tumours in hollow organs, as sharp and rigid cryoprobes can easily lead to organ perforation. The EBCA procedure can be directly observed and is safe and effective.^33^ In our previous studies, we confirmed the safety of EBCA in the bladder in beagle and porcine models.^15^, ^34^ The EBCA‐TUR study was the first multicentre randomised clinical trial to evaluate endoscopic cryoablation in patients with bladder cancer. In addition, we conducted a pioneer study of ureteroscopic cryoablation in patients with upper tract urothelial carcinoma of a solitary kidney as the first worldwide attempt to use cryotherapy as a kidney‐sparing strategy.^15^ Cryoablation significantly reduced recurrence and progression compared with traditional metronomic chemoablation, making our trial the first to report the application and promising outcomes of cryoablation in treating bladder cancer. Patients undergoing cryoablation have a good prognosis, which prompted us to further explore the underlying mechanisms.

In the present study, we first compared the size of the reimplanted secondary tumours and the prognosis after cryoablation and surgical resection of subcutaneous primary tumours in C57 mice and concluded that the secondary tumours grew more slowly and the mice survived longer after cryoablation. Based on previous studies on cryoablation releasing tumour antigens and activating T cells function, we further investigated the role of cryoablation in regulating T‐cell function and differentiation. Cryoablation not only promoted the infiltration of T cells into the tumour, turning it into an immune‐hot state, but also promoted the expansion of CD39^+^ tumour‐specific T cells and reduced the proportion of bystander T cells. However, further studies are required to demonstrate whether cryoablation transforms bystander T cells into tumour‐specific T cells, thereby contributing to a decrease in the proportion of bystander T cells. An innovative finding of this study is that the long‐lasting antitumour immune response induced by cryoablation was achieved by enhancing antitumour immune memory. We recorded the changes in the subpopulation of memory T cells in mice after cryoablation of the subcutaneous tumours and found that the proportion of T_cm_ and T_em_ cells started to increase approximately 14 days after cryoablation, while the proportion of T_cm_ and naïve T cells decreased significantly when the secondary tumours were implanted. These results suggest that these two subpopulations of T cells with differentiation and expansion abilities are activated and differentiate into effector T cells. In particular, we assessed the alterations in T_rm_ cells in tumour tissues. We observed that the proportion of T_rm_ cells significantly increased. This phenomenon provided direct evidence to validate the hypothesis that T_rm_ cells could differentiate from T_cm_ cells, reside in tissues, have antitumour properties, and perform secondary immune surveillance.

Another distinguishing feature of this study was the application of a coculture of tumour organoids with lymphocytes isolated from the same individual to evaluate the antitumour properties of lymphocytes. An organoid is a 3D cell culture differentiated and established in vitro, which can provide a physiologically relevant system by rebuilding a spatial structure similar to that of the original organ and imitating some of its functions. Compared with the traditional 2D cell culture model, tumour organoids can better simulate cellular differences within the tumour and mimic its reaction in specific physiological environments, such as hypoxia and immunosuppression. In vitro coculture experiments using tumour tissues and autologous peripheral blood collected before and after cryoablation not only reflected the regulation of the systemic immune response of the body and proved the stronger antitumour property of lymphocytes after cryoablation but also enabled further screening and expansion of tumour‐specific lymphocytes as a novel therapeutic method.

As recommended drugs for advanced metastatic bladder cancer, most PD‐1/PD‐L1 inhibitors benefit only 10%–50% of patients.^35^, ^36^, ^37^, ^38^ Therefore, it is necessary to explore combination treatment procedures that can enhance the efficacy of PD‐1/PD‐L1 inhibitors. We proposed and demonstrated that cryoablation combined with a PD‐1 inhibitor had a synergistic effect based on the elevated levels of antitumour effector molecules, such as IFN‐γ and immune checkpoints (e.g., PD‐1 after cryoablation). Physiologically, PD‐1 expression is initiated immediately after T‐cell activation to prevent overactivation. Therefore, the increase in immune checkpoints, such as PD‐1, after cryoablation does not reflect the exhaustion of T cells, but rather is a compensatory mechanism following T cell activation. We also dynamically examined the expression of immune checkpoints on day 1, day 3 and day 7 after cryoablation (Figure S6E) and found that the expression of immune checkpoints was downregulated in the early stage after cryoablation. However, PD‐1 (Pdcd1) showed a significant upregulation on day 7, while other effector molecules such as Prf1, Gzmb, Ifng and Tnf were also significantly upregulated at this time. In contrast, Havcr2, an indicator of T terminal depletion (TIM3), was indeed at a low expression. Thus, we hypothesised that T cells retain their strong antitumour properties even when the expression of PD‐1 is upregulated. Therefore, application of a PD‐1 inhibitor after cryoablation can have a long‐lasting, continuous, and powerful antitumour effect. Our hypothesis was proven in animal experiments, and more promisingly, we also found that combination therapy prolonged RFS of patients with high‐grade NMIBC and MIBC in our clinical study.

Although cryoablation performed a better clinical outcome, a few bladder cancer patients still relapse. There are several mechanisms for the failure of T cells to eliminate cancers, which eventually lead to tumour recurrence and metastasis. In addition to the effects of the TME‐mediated mechanisms, cancer cell‐mediated mechanism could not be ignored. In this study, we also attempted to identify some tumour intrinsic factors that might influence tumour recurrence after cryoablation. Among them, low expression of IFNGR (Interferon Gamma Receptor 1) was found to be associated with a higher risk of recurrence after cryoablation. IFNGR knocked out could render cryoablation‐induced tumour‐specific T cells ineffective in eliminating tumours. In addition, whether tumour intrinsic factors may induce recurrence and metastasis after cryoablation also deserves further investigation in our follow‐up study.

## 5 CONCLUSION

Endoscopic cryoablation eliminates tumour cells and releases tumour antigens, promoting long‐lasting tumour‐specific immunity to inhibit bladder tumour recurrence. Combining endoscopic cryoablation with ICIs offers new treatment options for patients with advanced bladder cancer.

## CONFLICT OF INTEREST STATEMENT

The authors have declared that no conflict of interest exists.

## Supporting information

Supporting Information
