# Supplementary material for: Cryoablation inhibits the recurrence and progression of bladder cancer by enhancing tumour‐specific immunity
Source: Clin Transl Med. 2023 May 8;13(5):e1255. doi: 10.1002/ctm2.1255 (PMC10167412; doi:10.1002/ctm2.1255)
Supplement: Supplementary file 1 — Supporting Information [file CTM2-13-e1255-s001.docx]

**Supplementary Information**

**Supplementary Figures**

**Supplementary Fig. 1**

**
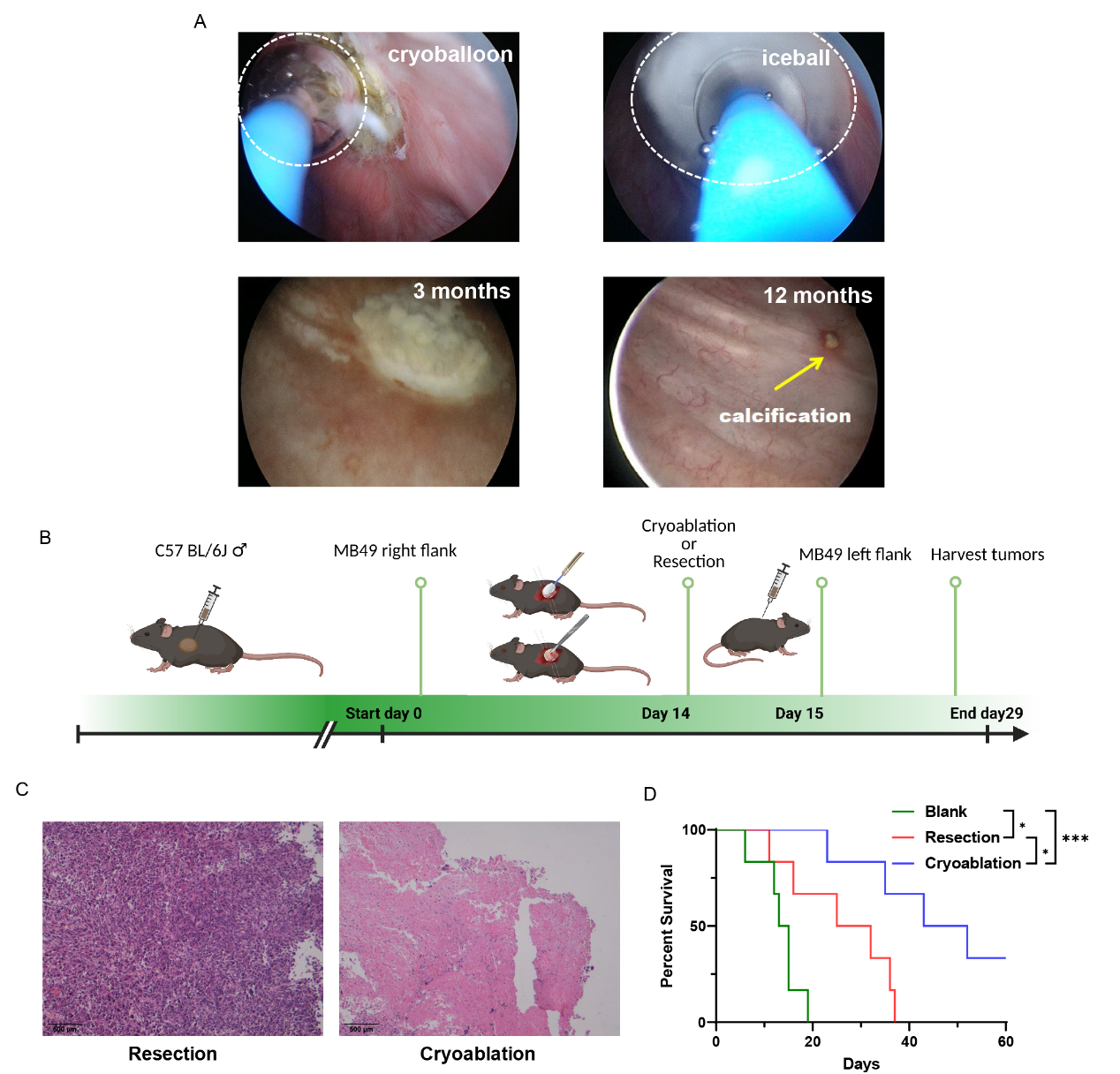
**

**(A)** Photos of endoscopic cryoablation procedure. A cryoballoon is used to form a therapeutic ice ball at the tumor site to achieve a tumor-killing effect. The tumor site was scarred and no recurrence was observed at three months and twelve months after the cryoablation respectively **(B)** Schematic diagram of the treatment of subcutaneous tumors by cryoablation or surgical resection and tumor rechallenge. **(C)** Effect of cryoablation on the subcutaneous tumor using immunohistochemical staining to compare the outcomes of cryoablation and surgical resection. (Right) After cryoablation, the cells were necrotic, with disrupted cellular structure and ruptured nuclei. Scale bars, 500μm. **(D)** Kaplan–Meier curves of the three groups of mice after rechallenge. n = 6 mice per group. **P* < 0.05, resection vs. blank and cryoablation; ****P* < 0.001, cryoablation vs. blank.

**Supplementary Fig.2
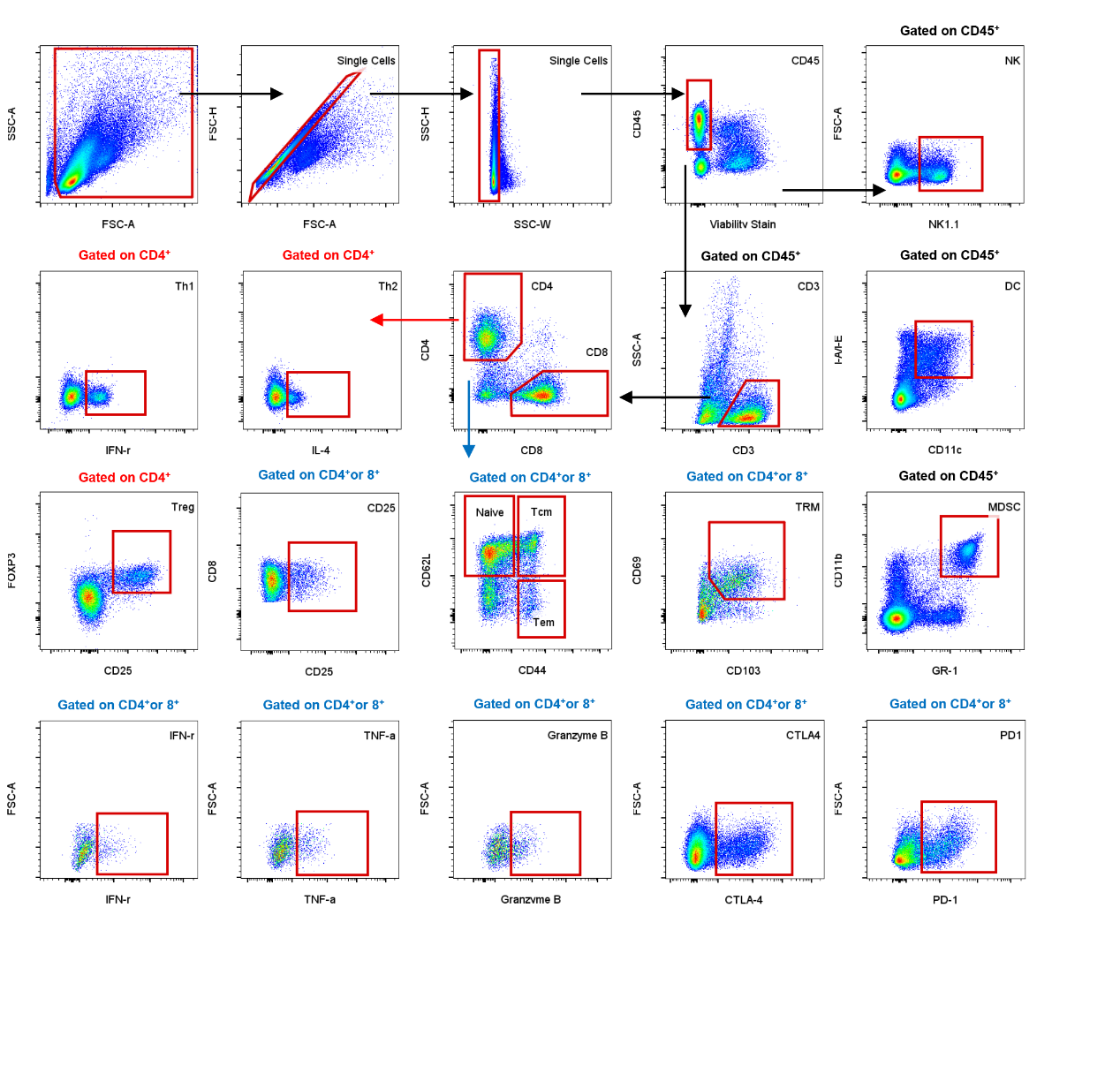
**

The total gating strategy for flow cytometry

**Supplementary Fig. 3**

**
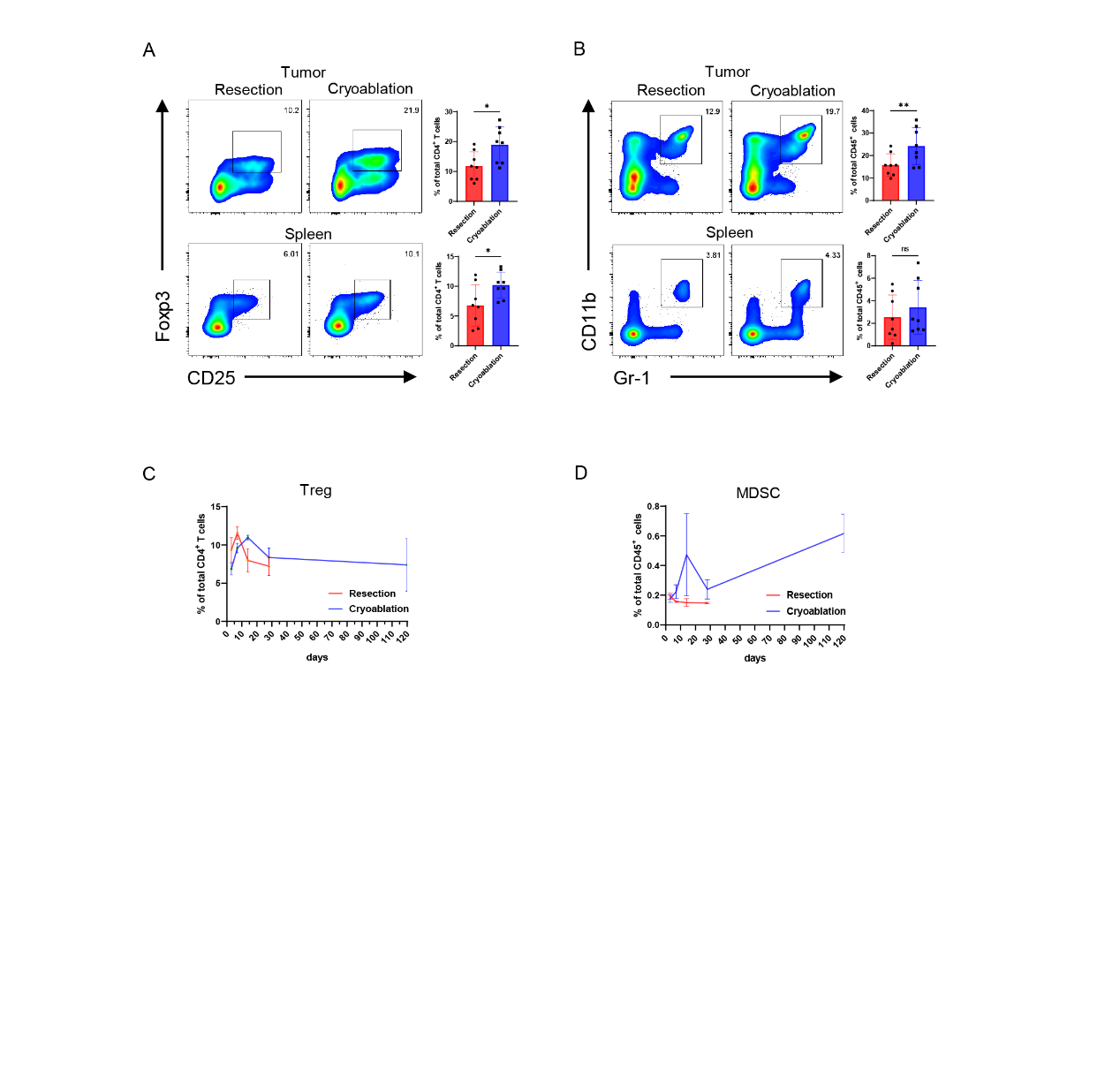
**

**(A)** Ratio of CD4^+^CD25^+^Foxp3^+^ Treg cells in secondary tumors and spleens of challenged mice after surgical resection or cryoablation of primary tumors measured by flow cytometry. n = 8 mice per group were observed. Data are represented as mean ± SD, **P*<0.05. **(B)** Ratio of CD11b^+^Gr-1^+^ MDSC in secondary tumors and spleens of challenged mice after surgical resection or cryoablation of primary tumors measured by flow cytometry. n = 8 mice per group. Data are represented as mean ± SD, ***P*<0.01. **(C-D)** The changing trends of Treg cells and MDSC in mice after resection or cryoablation of primary tumors via dynamic monitoring at day 3, 7, 14, 28, and 120.

**Supplementary Fig. 4**

**
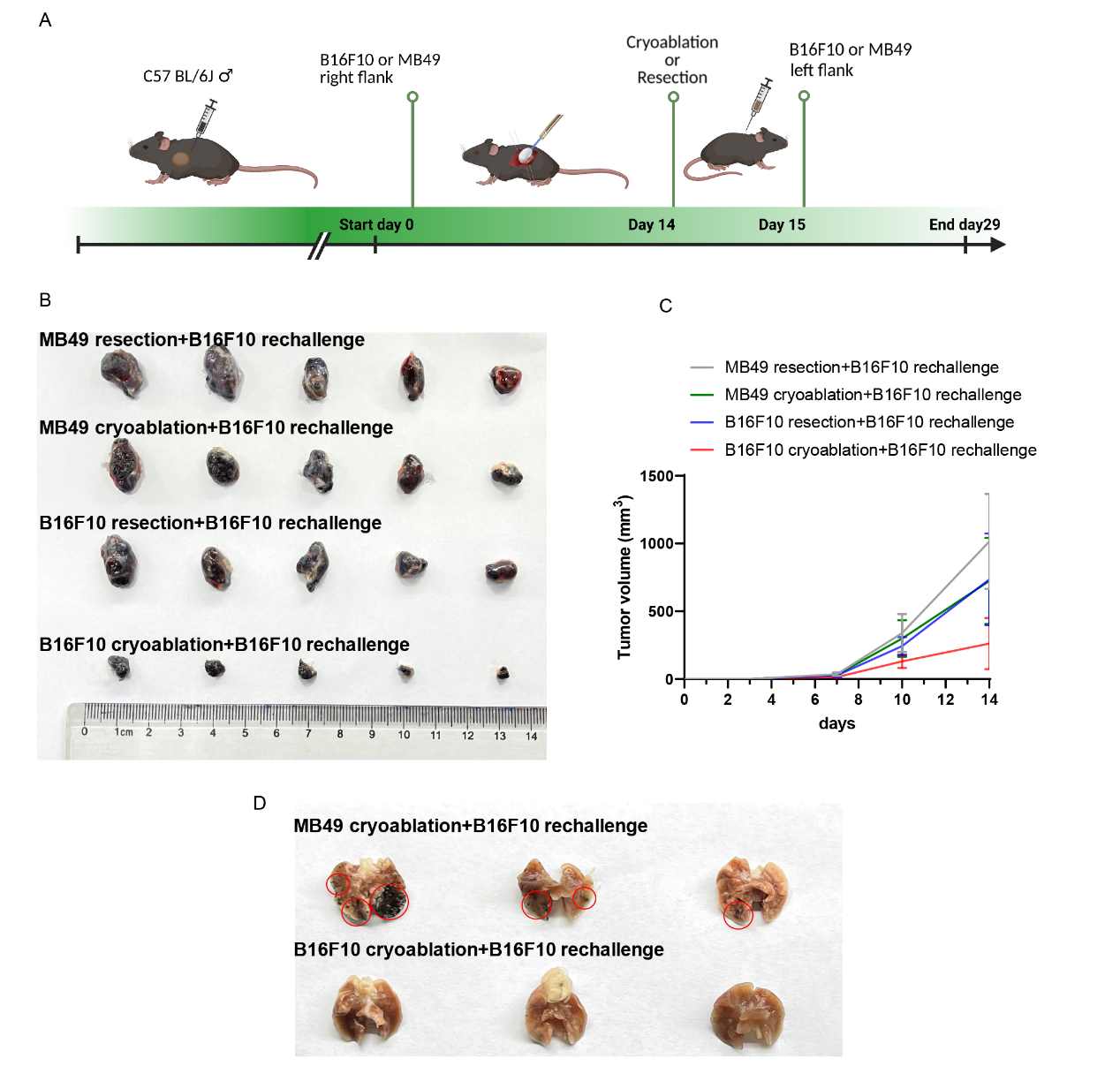
**

**(A)** Schematic illustrating the validation of tumor specificity of post-cryoablation immunity using B16F10 and MB49. **(B)** Secondary tumors were removed and measured after rechallenge with B16F10 cells on the second day after cryoablation or resection of the primary tumors. n = 5 mice per group. **(C)** Growth curve of contralateral secondary tumor. **(D)** Photographic images of lungs from B16F10 rechallenged on mice treated with MB49 or B16F10 cryoablation.

**Supplementary Fig. 5**

**
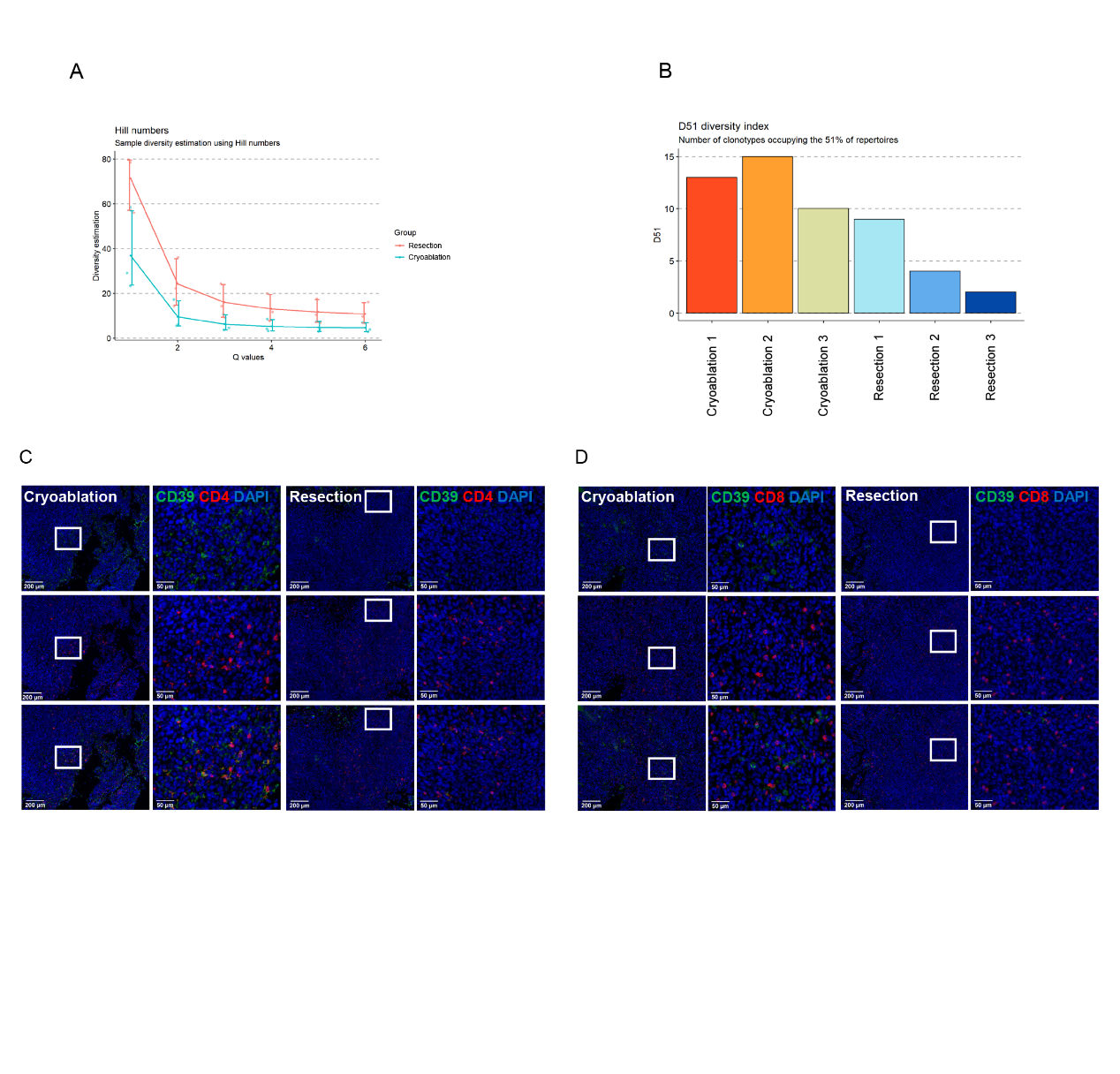
**

**(A, B)** TCR diversity quantified by Hill numbers(a), and D51diversity index(b). **(C, D)** Immunofluorescence staining of intratumor expression and distribution of CD39 (green) and CD4^+^/CD8^+^ (red) T cells. DAPI (blue) was applied for nuclear staining. Scale bars, 200μm (2x), Scale bars, 50μm (20x).

**Supplementary Fig. 6**

**
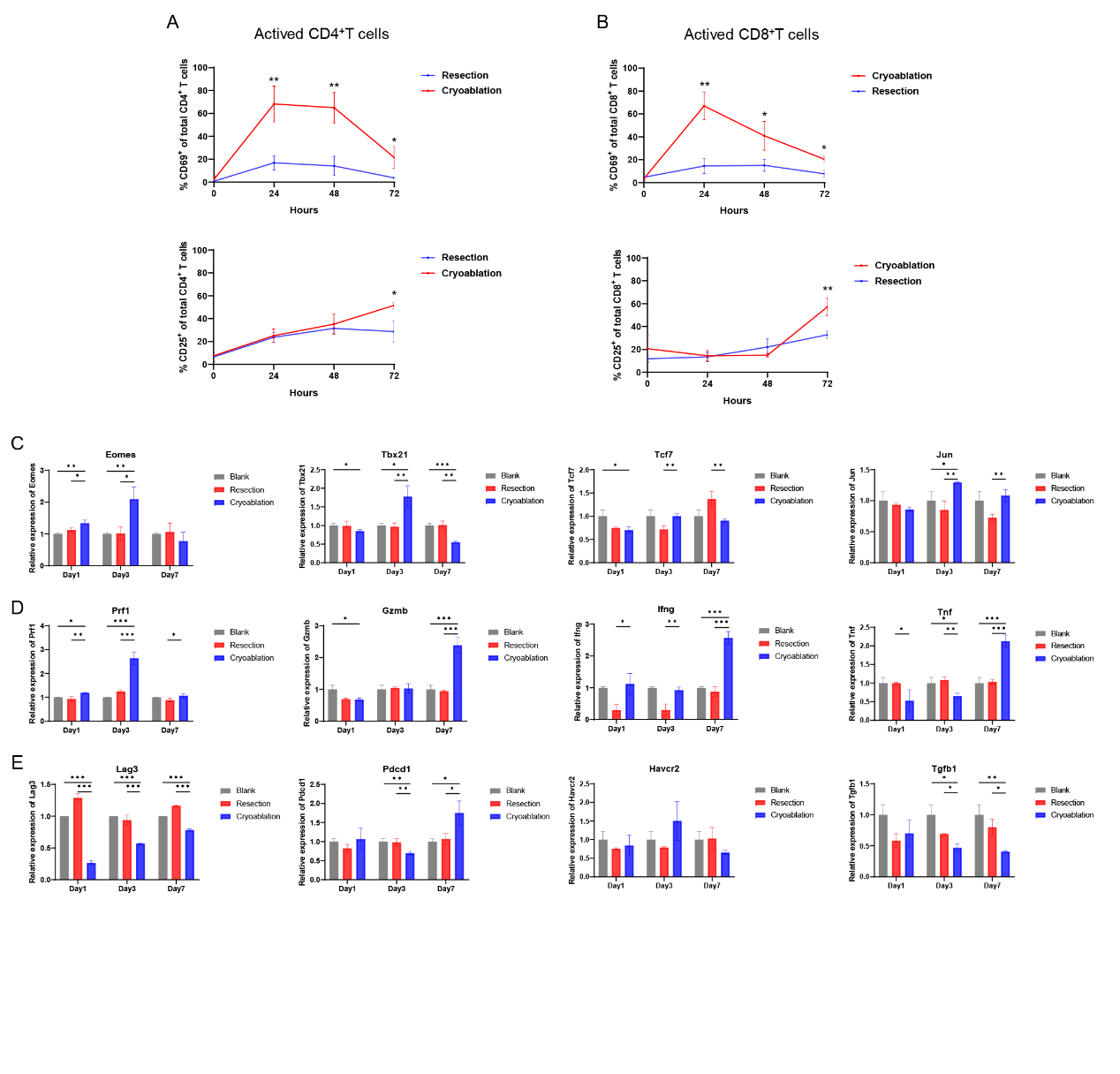
**

**(A, B)** Dynamic analysis of the subpopulation changes of CD69^+^ early active and CD25^+^ active CD4^+^ or CD8^+^ T cells by flow cytometry on 0h, 24h, 48h, 72h after cryoablation or resection. **P*< 0.05, ***P* < 0.01, Cryoablation vs. Resection **(C-E)** RNA Expression extracted from tumor of blank, resection and cryoablation groups detected by RT-qPCR. Data are presented as the mean ± SD. **P*< 0.05, ***P* < 0.01, ****P* < 0.001, Cryoablation vs. Blank or Resection.

**Supplementary Fig. 7**

**
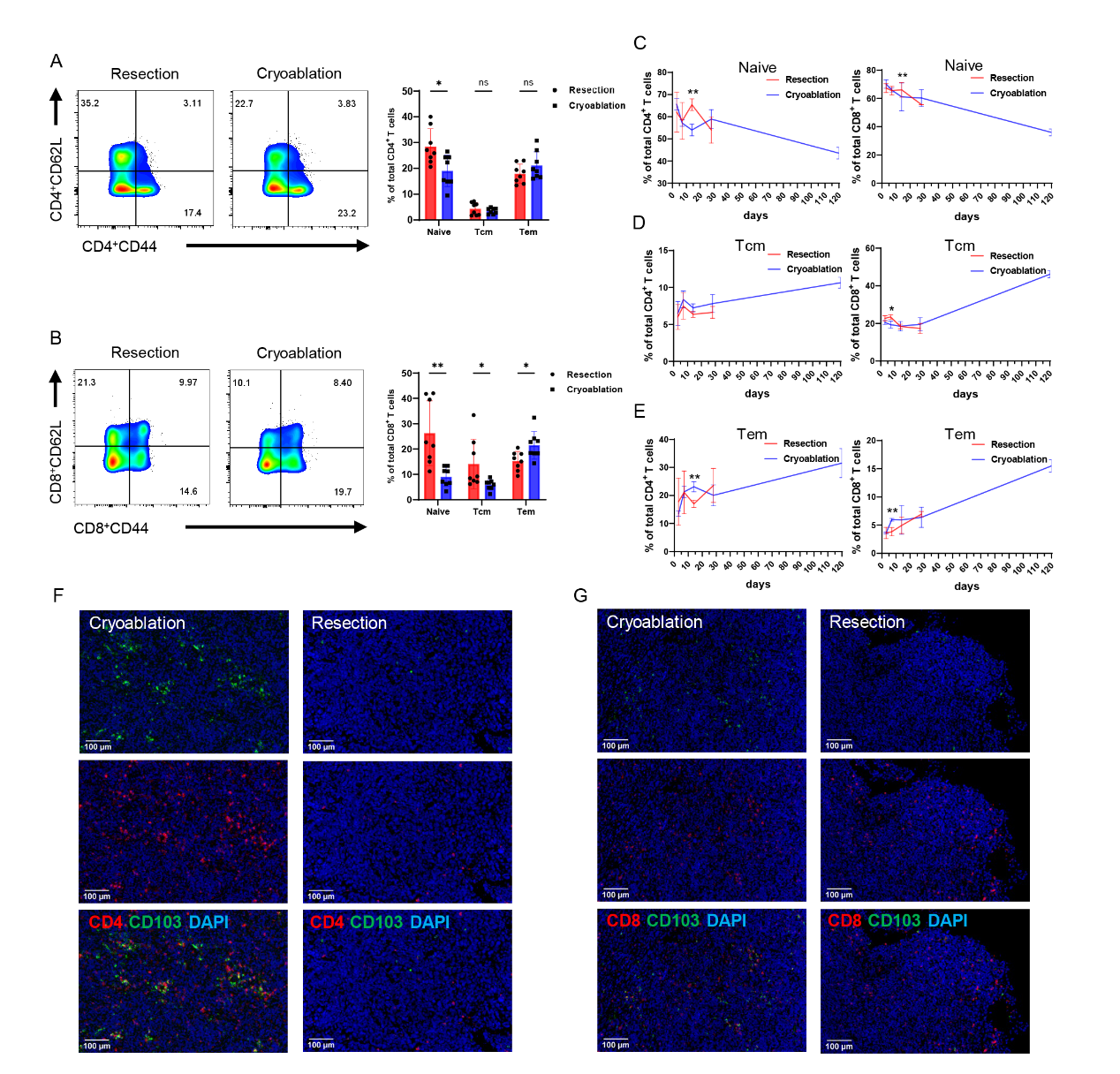
(A, B)** Flow cytometry showing the proportion of murine splenic CD4^+^ and CD8^+^ memory T lymphocytes in the cryoablation group (n = 8) and the resection group (n = 8) after rechallenge. CD62L^+^CD44^-^, naïve T cells; CD62L^+^CD44^+^, central-memory T cells; CD62L^-^CD44^+^, effector-memory T cells. Data are represented as mean ± SD, **P* < 0.05, ***P* < 0.01, cryoablation vs. resection. **(C-E)** Dynamic analysis of the subpopulation changes of memory T cells in the spleen of mice by flow cytometry on days 3, 7, 14, 28 and 120 after cryoablation or surgical resection. Three mice were examined in each group at each time. **(F, G)**. Spatial localization of the CD4^+^CD103^+^ and CD8^+^CD103^+^ double-positive cells in the tumor tissues by immunofluorescence, Scale bars, 100μm.

**Supplementary Fig. 8**

**
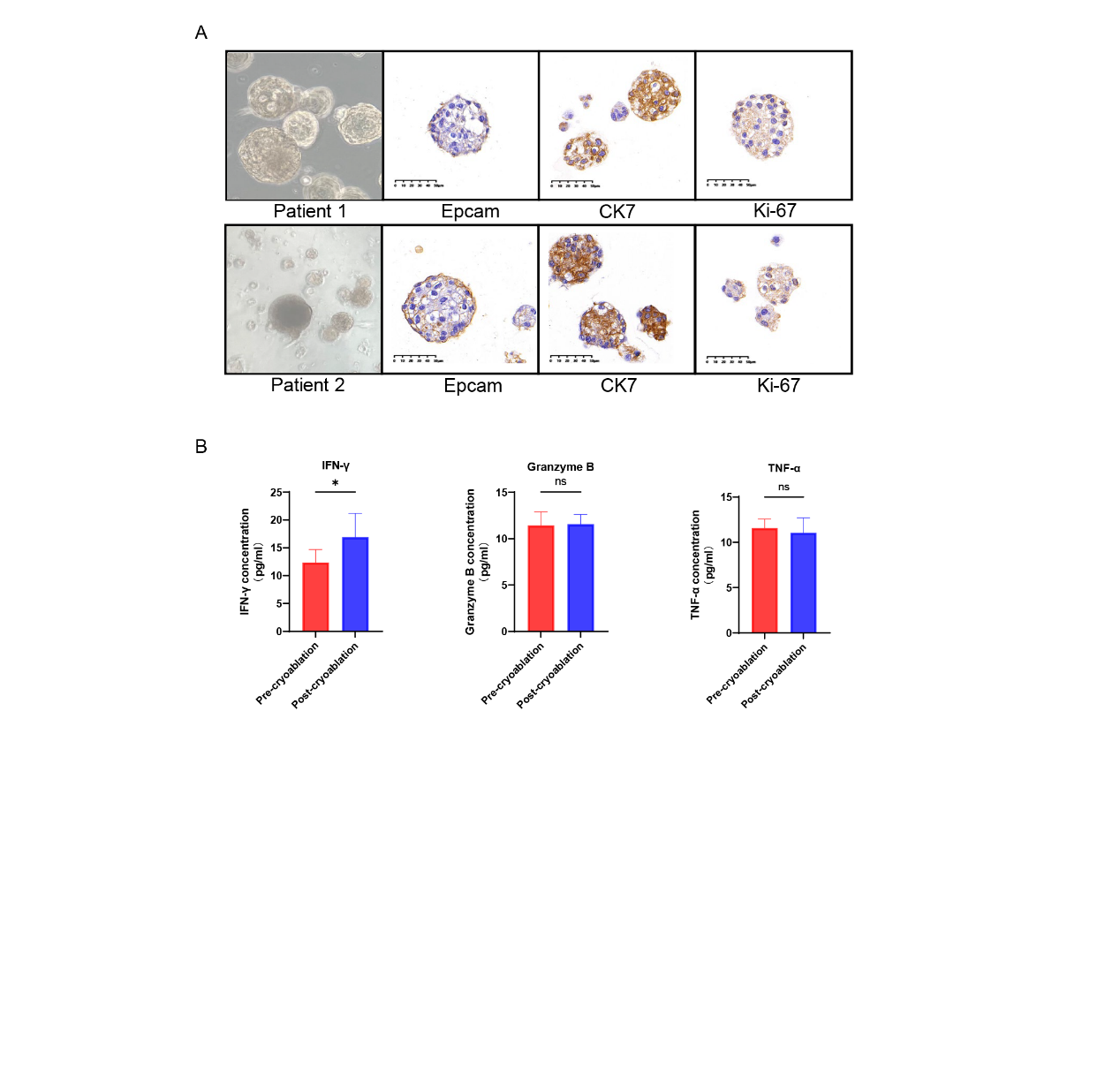
(A)** Bright-field images and immunohistochemical verification (Epcam, CK7 and Ki-67) of primary organoids derived from the tumor tissue of two patients who underwent cryoablation. Scale bars, 50 μm **(B)** ELISA showed cytokines collected after co-culture. Data are represented as mean ± SD, **P* < 0.05, post-cryoablation vs. pre-cryoablation.

**Supplementary Tables**

**Supplementary Table 1**

Cohort information in the prospective study evaluating cryoablation-based bladder sparing strategies

| **Characteristic** | **Cohort1** | |  | | **Corhort2** | |
| --- | --- | --- | --- | --- | --- | --- |
|  | **Cryoablation** | **Chemoablation** |  | **Cryoablation**  **+immunotherapy** | | **Cryoablation** |
| n | 38 | 30 |  | 13 | | 14 |
| Age, years |  |  |  |  | |  |
| Median | 65 | 63 |  | 65 | | 76 |
| Range | 33-83 | 31-78 |  | 50-81 | | 49-86 |
| Gender, n (%) |  |  |  |  | |  |
| Female | 7(18) | 5(17) |  | 5(38) | | 1(7) |
| Male | 31(82) | 25(83) |  | 8(62) | | 13(93) |
| NMIBC, n (%) | 37(97) | 29(97) |  | 7(54) | | 11(79) |
| MIBC, n (%) | 1(3) | 1(3) |  | 6(46) | | 3(21) |
| Tumor grade, n (%) |  |  |  |  | |  |
| PUNLMP | 2(5) | 1(3) |  | / | | / |
| Low-grade | 24(63) | 18(60) |  | 2(15) | | / |
| High-grade | 12(32) | 11(37) |  | 11(85) | | 14(100) |
| Tumor number, n (%) |  |  |  |  | |  |
| Single | 23(61) | 18(60) |  | 6(46) | | 5(36) |
| Multiple | 15(39) | 12(40) |  | 7(54) | | 9(64) |
| Maximal tumor size, cm |  |  |  |  | |  |
| Median | 2 | 2 |  | 2 | | 3 |
| Range | 0.5-3 | 0.3-3 |  | 0.5-5 | | 0.5-4 |

**Supplementary Table 2**

Information of antibodies used for flow cytometric analysis

| **Antibodies** | **Clone** | **Source** |
| --- | --- | --- |
| FITC anti-mouse CD3 | 145-2c11 | Biolegend |
| PE anti-mouse CD4 | GK1.5 | Biolegend |
| Brilliant Violet 510™ anti-mouse CD4 | RM4-5 | Biolegend |
| APC-eFluor 780 anti-mouse CD8a | 53-6.7 | eBioscience |
| PE/Cyanine7 anti-mouse/human CD44 | IM7 | Biolegend |
| PerCP/Cyanine5.5 anti-mouse CD45 | 30-F11 | Biolegend |
| PE/Cyanine7 anti-mouse CD45 | 30-F11 | Biolegend |
| APC anti-mouse CD62L | MEL-14 | Biolegend |
| APC anti-mouse CD103 | 2.00E+07 | Biolegend |
| eFluor 450 anti-mouse CD69 | H1.2F3 | eBioscience |
| PE anti-mouse IFN-r | XMG1.2 | eBioscience |
| PE/Cyanine7 anti-mouse NK1.1 | S17016D | Biolegend |
| PE/Cyanine7 anti-mouse CD45R (B220) | RA3-6B2 | eBioscience |
| PE/Cyanine7 anti-mouse CD80 | 16-10A1 | Biolegend |
| APC anti-mouse Granzyme B | NGZB | eBioscience |
| PE/Cyanine7 anti-mouse TNF-a | MP6-XT22 | eBioscience |
| eFluor 450 anti-mouse PD-1 | J43 | eBioscience |
| PE/Cyanine7 anti-mouse CD152 | UC10-4B9 | Biolegend |
| APC anti-mouse CD11b | M1/70 | eBioscience |
| Brilliant Violet 421™ anti-mouse Ly6G/6C | RB6-8C5 | Biolegend |
| Brilliant Violet 421™ anti-mouse CD86 | GL-1 | Biolegend |
| APC anti-mouse CD11c | N418 | Biolegend |
| PE anti-mouse MHCII (I-A/I-E) | M5/114.15.2 | Biolegend |
| Alexa Fluor 647 anti-mouse IL-4 | 11B11 | Biolegend |
| APC anti-mouse Foxp3 | 3G3 | TONBO |
| PE anti-mouse CD25 | PC61.5 | eBioscience |
| BV421 anti-mouse CD119 (IFNGR) | GR20 | BD pharmingen |
| APC anti-human CD197(CCR7) | G043H7 | Biolegend |
| PE/Cyanine7 anti-human CD45RA | HI100 | Biolegend |
| PE anti-human CD45RO | UCHL1 | Biolegend |
| APC/Fire™750 anti-human CD4 | RPA-T4 | Biolegend |
| FITC anti-human CD8a | HIT8a | Biolegend |
| PerCP/Cyanine5.5 anti-human CD3 | UCHT1 | Biolegend |
| PerCP anti-human CD8a | SK1 | Biolegend |
| FITC anti-human CD4 | SK3 | Biolegend |
| Brilliant Violet 510™ anti-human CD45 | HI30 | Biolegend |

**Supplementary Table 3**

Primer list of RT-qPCR:

| Name | Sequence (5'to3') | |
| --- | --- | --- |
| Tbx21 | F: TGTTCCCAGCCGTTTCTACC | R: GCTCGGAACTCCGCTTCATA |
| Eomes | F: CTAGGGGAATCCGTGGGAGA | R: TGTGACGGCCTACCAAAACA |
| Jun | F: GCACATCACCACTACACCGA | R: GGGAAGCGTGTTCTGGCTAT |
| Tcf7 | F: GTGAATGAGTCCGAAGGCG | R: AGTCTCTGCGAAGTGTGCTC |
| Ifng | F: AGCAAGGCGAAAAAGGATGC | R: TCATTGAATGCTTGGCGCTG |
| Gzmb | F: ACAACACTCTTGACGCTGGG | R: CGAGAGTGGGGCTTGACTTC |
| Prf1 | F: TCTTGGTGGGACTTCAGCTT | R: ATTCTGACCGAGTGGCAGTG |
| Tnf | F: TAGCCCACGTCGTAGCAAAC | R: ACAAGGTACAACCCATCGGC |
| Pdcd1 | F: TCCAACTGGTCGGAGGATCT | R: GTATGATCTGGAAGCGGGCA |
| Havcr2 | F: GGGTTGACCCTGGCACTTAT | R: TCAGAGCGAATCCTGACTGC |
| Lag3 | F: AGGCCATCTCGTTCTCGTTC | R: CCGGAAATGGCTGAATCCCA |
| Tgfb1 | F: TTGTACGGCAGTGGCTGAAC | R: TTTGGGGCTGATCCCGTTGA |
| Gapdh | F: AATGGTGAAGGTCGGTGTGA | R: TGAGGTCAATGAAGGGGTCG |
